# Biofilm formation by clinical isolates and the implications in chronic infections

**DOI:** 10.1186/1471-2334-13-47

**Published:** 2013-01-29

**Authors:** Carlos J Sanchez, Katrin Mende, Miriam L Beckius, Kevin S Akers, Desiree R Romano, Joseph C Wenke, Clinton K Murray

**Affiliations:** 1 Department of Extremity Trauma and Regenerative Medicine, United States Army Institute of Surgical Research, Ft. Sam Houston, San Antonio, TX, USA; 2 Infectious Disease Clinical Research Program, Uniformed Services University of the Health Sciences, Bethesda, MD, USA; 3 Department of Medicine, Infectious Disease Service, San Antonio Military Medical Center, Ft. Sam Houston, San Antonio, TX, USA; 4 Department of Clinical Investigation, San Antonio Military, Medical Center, Ft. Sam Houston, San Antonio, TX, USA

**Keywords:** Biofilm formation, Clinical isolates, Chronic infection, Multidrug-resistant, MRSA

## Abstract

**Background:**

Biofilm formation is a major virulence factor contributing to the chronicity of infections. To date few studies have evaluated biofilm formation in infecting isolates of patients including both Gram-positive and Gram-negative multidrug-resistant (MDR) species in the context of numerous types of infectious syndromes. Herein, we investigated the biofilm forming capacity in a large collection of single patient infecting isolates and compared the relationship between biofilm formation to various strain characteristics.

**Methods:**

The biofilm-forming capacity of 205 randomly sampled clinical isolates from patients, collected from various anatomical sites, admitted for treatment at Brooke Army Medical Center (BAMC) from 2004–2011, including methicillin-resistant/methicillin susceptible *Staphylococcus aureus* (MRSA/MSSA) (n=23), *Acinetobacter baumannii* (n=53), *Pseudomonas aeruginosa* (n=36), *Klebsiella pneumoniae* (n=54), and *Escherichia coli* (n=39), were evaluated for biofilm formation using the high-throughput microtiter plate assay and scanning electron microscopy (SEM). Relationships between biofilm formation to clonal type, site of isolate collection, and MDR phenotype were evaluated. Furthermore, in patients with relapsing infections, serial strains were assessed for their ability to form biofilms *in vitro*.

**Results:**

Of the 205 clinical isolates tested, 126 strains (61.4%) were observed to form biofilms *in vitro* at levels greater than or equal to the *Staphylococcus epidermidis*, positive biofilm producing strain, with *P. aeruginosa* and *S. aureus* having the greatest number of biofilm producing strains. Biofilm formation was significantly associated with specific clonal types, the site of isolate collection, and strains positive for biofilm formation were more frequently observed to be MDR. In patients with relapsing infections, the majority of serial isolates recovered from these individuals were observed to be strong biofilm producers *in vitro*.

**Conclusions:**

This study is the first to evaluate biofilm formation in a large collection of infecting clinical isolates representing diverse types of infections. Our results demonstrate: (1) biofilm formation is a heterogeneous property amongst clinical strains which is associated with certain clonal types, (2) biofilm forming strains are more frequently isolated from non-fluid tissues, in particular bone and soft tissues, (3) MDR pathogens are more often biofilm formers, and (4) strains from patients with persistent infections are positive for biofilm formation.

## Background

Multidrug-resistant (MDR) organisms, including *Acinetobacter baumannii*, methicillin-resistant *Staphylococcus aureus* (MRSA), and extended spectrum beta-lactamase (ESBL) producing Gram-negative bacteria, are frequently implicated as the causative agents of acute and chronic infections contributing significantly to patient morbidity and mortality, as well as increased health care costs associated with treatment [1,2]. Numerous studies to date indicate that human infections are, in large part, caused by the ability of bacteria to develop surface attached polymicrobial communities known as biofilms [3-5]. Microbial biofilms consist of groups of bacterial cells adherent to a surface and enclosed within a self produced extracellular matrix [6]. Adaptation to surface attached growth within a biofilm is accompanied by significant changes in gene and protein expression, as well as metabolic activity [7,8] which confers resistance to antimicrobial therapy [9,10] and host mechanisms of clearance [11,12]. Many pathogenic and nosocomial bacteria have been observed to predominantly exist as biofilms, in both natural environments and within infected tissues as polymicrobial communities [4,5,13-15]. Importantly, biofilm formation is implicated as a significant factor involved in a number of chronic human infections [4,16,17].

Although there are numerous studies to date describing the ability of clinical strains to form biofilms *in vitro* (Additional file 1), these have varied widely in their study design, method of characterizing isolates and their biofilms, strains and number of isolates used, and most have omitted molecular assessments of strain relatedness to ensure a genetically heterogeneous sample and link biofilm production to strain type, with the exception of few studies evaluating biofilm formation and SCC*mec* and *spa* typing in staphylococci [18-22]. Likewise, few studies have surveyed biofilm formation among Gram-negative clinical isolates outside the context of genitourinary tract infections [23,24]. Furthermore, to our knowledge although biofilms have been implicated to be involved in chronic infections, no studies have characterized biofilm production from isolates recovered from relapsed infections where the clonal identity was proven identical to the initial infecting strains. Herein, we evaluated the biofilm forming capacity in a large collection of single-patient bacterial isolates, representing both Gram-positive and Gram-negative bacterial species, recovered from patients admitted for treatment at our medical facility for numerous types of infectious syndromes. Additionally, the biofilm formation phenotype was evaluated in the context of relapsing infection, where serial isolates were available for study.

## Methods

### Bacterial isolates and growth conditions

The 205 clinical isolates used in this study were selected from a strain repository at Brooke Army Medical Center (BAMC; Fort Sam Houston, TX, USA). Bacterial strains from the repository were collected from patients as part of the standard care and infection control policy not related to research from 2004–2011. The 205 single and serial clinical isolates were randomly selected from the strain collection representing a total of 150 patients and multiple anatomical collection sites, including wound cultures, bone, respiratory tract, urine and blood (Table 1). As a positive control for biofilm formation, the previously characterized biofilm forming strain *S. epidermidis* ATCC strain 12228 was used [25,26]. Bacterial cultures were frozen and maintained at −80°C and sub-cultured on blood agar plates (Remel, Lenexa, KS) overnight at 37°C prior to each experimental assay. With the exception of *S. aureus*, which was cultured in tryptic soy broth (TSB), all bacteria were grown in Luria-Bertani broth (LB) overnight at 37°C. Antimicrobial susceptibility testing for all strains was performed using the BD Phoenix™ automated microbiology system as recommended by the manufacturer (BD, Franklin Lakes, NJ). Resistance and susceptibilities to the various drugs tested are reflective of the clinical breakpoints set by the Clinical and Laboratory Standards Institute (CLSI) as described by the performance standards for antimicrobial susceptibility testing (M100-S22, Jan 2012).

**Table 1:** Characteristics of clinical isolates used in this study

**Bacterial species**	**Clinical isolates**	**# of patients**	**Pulsed- Field Type (PFTs)**	*Phenotype	**Site of isolation**
*E. coli*	39	32	1 (n=4)	ESBL+ (n=31)	Wound culture (n=10)
			2 (n=5)		Blood (n=4)
			3 (n=3)		Urine (n=21)
			4 (n=7)		
			7 (n=5)		
			Other (n=15)		
*K. pneumoniae*	54	33	1 (n=6)	MDR (n=54)	Wound culture (n=39)
			2 (n=8)		Blood (n=10)
			3,4,14,16,17, 18 (n=5)		Respiratory (n=5)
			Other (n=10)		
*P. aeruginosa*	36	17	1 (n=7)	MDR (n=28)	Wound culture (n=29)
			2, 18 (n=5)		Blood (n=7)
			Other (n=19)		
*A. baumannii*	53	47	1 (n=13)	MDR (n=46)	Wound culture (n=31)
			5 (n=4)		Blood (n=20)
			2,3,4, 6,7,14 (n=5)		Urine (n=1)
			Other (n=6)		Respiratory (n=1)
*S. aureus*	23	21	USA100 (n=10)	MRSA (n=15)	Wound culture (n=14)
			USA200, USA800 (n=4)	MSSA (n=8)	Blood (n=4)
			USA300 (n=2)		Respiratory (n=5)
			USA700 (n=3)		

*A multidrug-resistant (MDR) organism was defined as any extended spectrum beta-lactamase (ESBL)-producing bacteria, or if resistant to all tested antimicrobials in 3 or more classes of antimicrobial agents (penicillins/cephalosporins, carbapenems, aminoglycosides, and quinolones) not including tetracyclines or colistin.

### Pulsed-field gel electrophoresis (PFGE)

Clonal relationships between bacterial strains of each individual species were assessed by pulsed-field gel electrophoresis (PFGE) according to the FDA method ‘Procedure for PFGE of Gram-negative rods’ (Version 1, 10/30/2007) and as previously described using the CHEF-DRIII system (Bio-Rad Laboratories, Hercules, California) [27,28]. The endonuclease *ApaI* was used for *A. baumannii* PFGE, *SmaI* for MRSA PFGE, *XbaI* for *K. pneumoniae* and *E. coli* PFGE and *SpeI* was used for *P. aeruginosa* PFGE. Gel images were analyzed using BioNumerics software (Applied Maths, Austin, TX). PFGE patterns were interpreted and grouped into pulsed field types (PFTs) using previously established criteria [27,29].

### Biofilm formation in 96-well microtiter plates

Biofilm formation was examined by the semi-quantitative determination of biofilm formation in 96-well flat bottom plates as previously described [30,31]. Briefly, fresh bacterial suspensions were prepared in either TSB or LB from overnight cultures and adjusted to OD_600_ of 0.1 (~ 10^7^ CFU/mL). 100 μL aliquots of bacterial suspension were then inoculated into individual wells of a 96-well flat-bottomed polystyrene plate and incubated overnight at 37°C for 48h. Following overnight incubation, plates were gently washed with 1X phosphate buffered saline (PBS; pH 7.4) and stained with 100 μL of 0.1% Crystal Violet (Sigma-Aldrich, St. Louis, MO) for 30 min at room temperature. Excess crystal violet was removed by washing, and biofilm was quantified by measuring the corresponding OD_570 nm_ of the supernatant following the solubilization of CV in 95% ethanol. For each clinical strain tested, biofilm assays were performed in triplicate and the mean biofilm absorbance value was determined. Strains that formed biofilms ≥ OD_570_ of the positive control were considered to be positive for biofilm formation whereas those strains with values less than the control were considered as weak biofilm forming strains.

### Visualization of biofilms by Scanning-Electron Microscopy (SEM)

A single representative strain from each of the individual bacterial species demonstrating significant biofilm formation based on the microtiter plate assay, were further characterized by SEM. Bacterial biofilms were grown on polystyrene pegs using the MBEC™-P&G plates (Innovotech, Alberta, Canada) for 48h, following the methods described above, and SEM was performed using previously described optimized conditions [32,33]. Briefly, following incubation, pegs were rinsed with 1X PBS and removed using sterile needle-tipped pliers. Each peg was then fixed with 2% (w/v) glutaraldehyde, 2% (w/v) paraformaldehyde, 0.15 M sodium cacodylate, 0.15% (w/v) alcian blue for 3 hr at room temperature. Pegs were then rinsed three times with 0.15 M sodium cacodylate buffer, immersed in 1% (v/v) osmium tetroxide in sodium cacodylate and incubated for 1 hr at room temperature. Pegs were then rinsed three times with distilled water followed by a stepwise dehydration with ethanol (i.e. 70%, 95%, and 100%). Samples were then treated with hexamethyldisilizane for 5 min prior to drying in a desiccator overnight. Next day samples were sputter coated with gold palladium and viewed with a JEOL-6610 scanning electron microscope. SEM experiments were carried out in duplicate for each strain tested, and representative images of biofilms were selected.

### Statistical analysis

Statistical analysis was performed using One-way ANOVA with a Holm-Sidak post-hoc evaluation for comparison between multiple groups. For non-parametric comparisons between groups, the *Χ*^*2*^ test was performed. *P* values of <0.05 were considered to be statistically significant.

## Results and discussion

### Clinical strains have a heterogeneous capacity for biofilm formation

**Figure 1:**
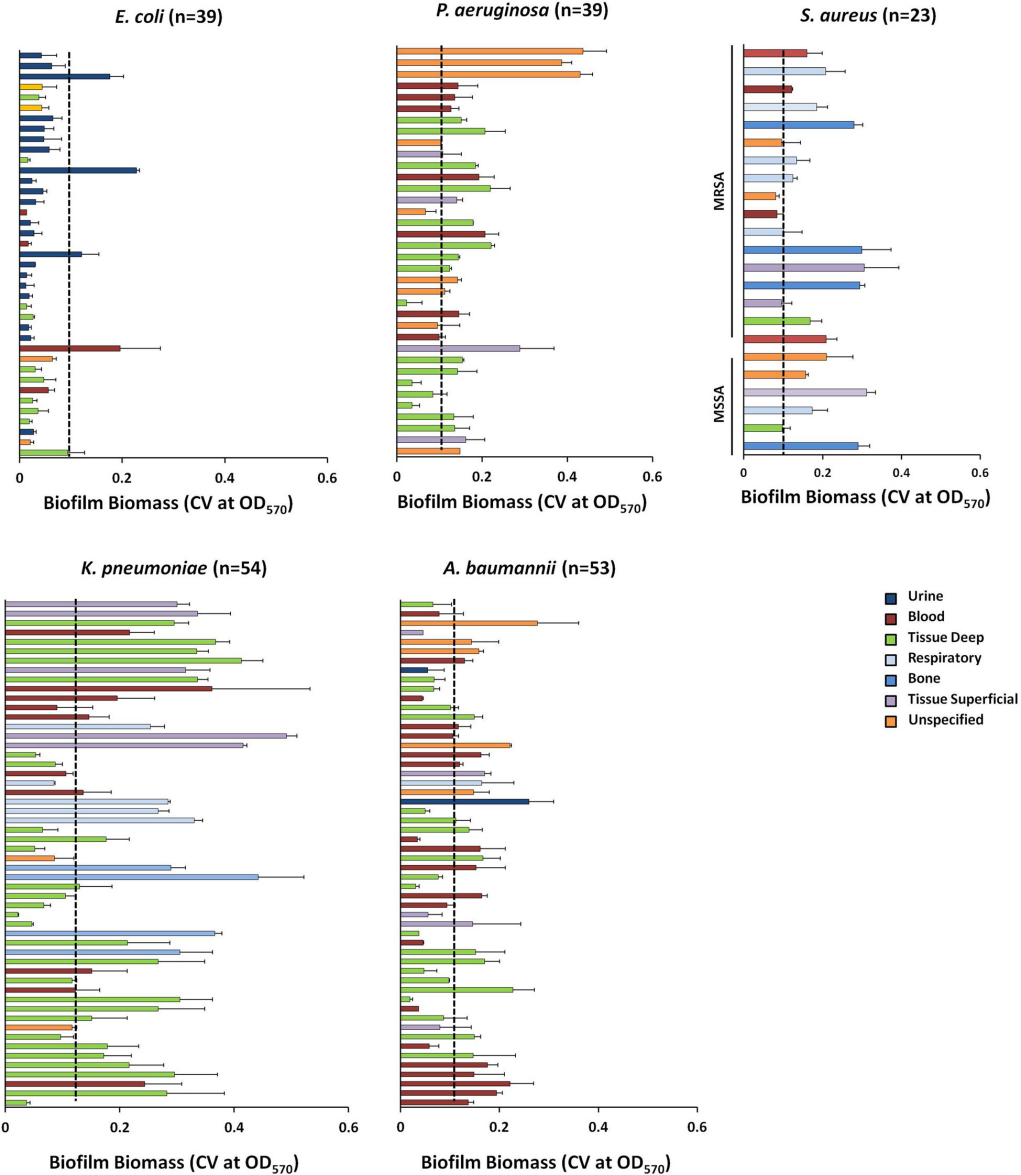
**Biofilm formation by clinical bacterial isolates.** Biofilm formation by individual clinical isolates of *E. coli* (n=39), *P. aeruginosa* (n=36), *S. aureus* (n=23), *K. pneumoniae* (n=54), and *A. baumannii* (n=53) using the 96-well microtiter plate assay. Biofilm formation was assessed by staining the attached bacteria with 0.1% CV and measuring the OD values at 570nm (CV_570_) after 48 h growth at 37°C. Bars are representative of the average biofilm biomass from three independent experiments for each clinical isolate tested. Error bars indicate the standard error. Dashed line (−−-) indicates average biofilm biomass value (OD_570_ = 0.122) for *S. epidermidis* ATCC 12228, the positive control for biofilm formation. Bars representing individuals strains are color coded to indicate site of isolation. Clinical isolates were ordered randomly with the exception of *S. aureus* which was separated by methicillin resistant (MRSA) and sensitive (MSSA) strains.

The ability of clinical isolates to form biofilms is associated with the capacity of these organisms to survive within hospital environments, on implanted medical devices, and in the wounds of patients [4,16,17]. The majority of studies examining biofilm formation in clinical isolates to date have focused primarily on isolates representative of bacterial species associated with device related infections (Additional file 1). Consequently, limited studies on biofilm formation in certain bacterial species, including *A. baumannii* and *K. pneumoniae*, associated with other clinical diseases are available. To address this, we evaluated the ability of individual clinical isolates from a diverse collection of infecting isolates to develop biofilms using the semi-quantitative 96-well plate assay as described [30,34,35]. This static model of biofilm formation has been demonstrated to be a reliable and a reproducible method for assessing biofilm formation *in vitro*. Of the 205 clinical strains evaluated for biofilm formation, more than half of all isolates (61.4%; 126/205) were observed to be capable of forming biofilms equal to or greater than the biofilm control strain, *S. epidermidis* ATCC 12228 (Figure 1). As shown in Figure 1, biofilm formation by individual isolates was heterogeneous and dependent on both the bacterial species and strain.

Of the 23 *S. aureus* isolates tested, 21 strains (91%) were positive for biofilm formation, with a median biofilm biomass of 0.121 ± 0.074 and 0.161 ± 0.072 for MRSA and MSSA, respectively. Consistent with previous reports, no significant differences in biofilm formation between the MRSA and MSSA strains were observed (*p*=0.40), indicating no significant correlation between methicillin susceptibility and the ability to form biofilms [21,22]. In *K. pneumoniae*, 41 of the 54 isolates tested (76%) were determined to be positive for biofilm formation with a median biofilm biomass of 0.214 ± 0.118. Likewise, in *P. aeruginosa* and *A. baumannii*, 30 of 36 (83%) and 29 of 53 (55%) of clinical strains tested were observed to form biofilms with a median biofilm biomass of 0.142 ± 0.094 and 0.125 ± 0.061, respectively. In contrast, *E. coli* was the weakest biofilm forming group with only 5 of the 39 strains (13%) capable of forming biofilms greater than the control strain and having the lowest biofilm biomass (median 0.044 ± 0.018). As the majority of *E. coli* strains (64.2%) were collected from fluid sites, blood (10.2%; 1 biofilm positive) and urine (54%; 3 biofilm positive), and those strains positive for biofilm formation were primarily from these sites, the low prevalence of biofilm formation observed herein is likely the result of the random sampling from our collection as well as the strain types represented, discussed below, as previous studies have demonstrated that clinical isolates of *E. coli* are capable of forming biofilms *in vitro*[23]. Importantly for those strains that were observed to be poor biofilm formers *in vitro*, these strains may still be important during polymicrobial infections where they can directly be incorporated into an established biofilm or interact with other species providing synergy to the biofilm formers.

**Figure 2:**
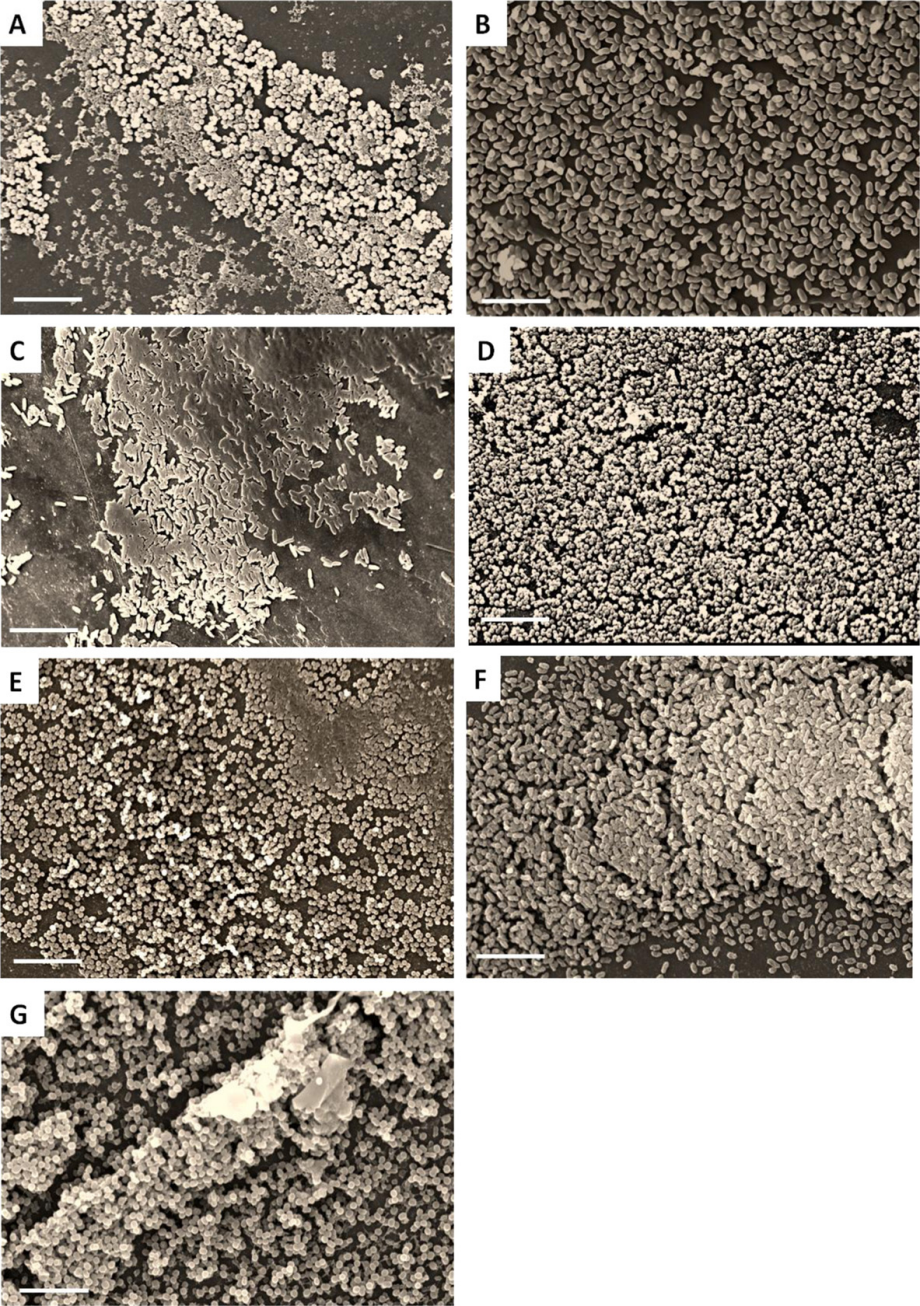
**Scanning Electron Microscopy (SEM) images of biofilms.** Representative SEM images of biofilms established on polystyrene pegs following 48 h incubation at 37°C from a selected biofilm producing strain of each bacterial species; including **A**) *S. epidermidis* ATCC 12228 (positive control), **B**) *E. coli*, **C**). *P. aeruginosa,***D**) *S. aureus* (MRSA), **E**) *S. aureus* (MSSA), **F**) *K. pneumoniae*, and **G**) *A. baumannii.* SEM pictures were taken at 2000X magnification; inset white bar is representative of 10 microns.

As confirmation of biofilm formation using the 96 well plate model, we examined biofilm formation by scanning electron microscopy (SEM) using the MBEC™-P&G plates as previously described [32,33]. For this study, single representative isolates from each species positive for biofilm formation, as determined by the microtiter plate method, were selected for SEM analysis. In agreement with the results from the microtiter plate assay, SEM studies demonstrated that the strains selected were capable of forming mature, robust biofilms on the peg surface, albeit the mature biofilm structures and phenotypes observed were unique for each species (Figure 2A-G). Together these findings demonstrate that biofilm formation is a prevalent feature amongst clinical strains associated with numerous infectious syndromes. 

### Relationship of biofilm formation to pulsed-field type and culture site

The 205 isolates tested for biofilm formation herein represented >29 unique PFTs in the five different bacterial species, which were representative of the range of clinical strains encountered within our healthcare treatment facility. The pathogenic potential of numerous organisms has been linked to the PFT. For example, MRSA USA300 has been implicated in outbreaks within the US, accounting for up to 70% of all skin and soft tissue infections [36,37]. Given the relationship between PFT and strain virulence, we evaluated whether a similar association between biofilm formation and PFT could be made.

**Figure 3:**
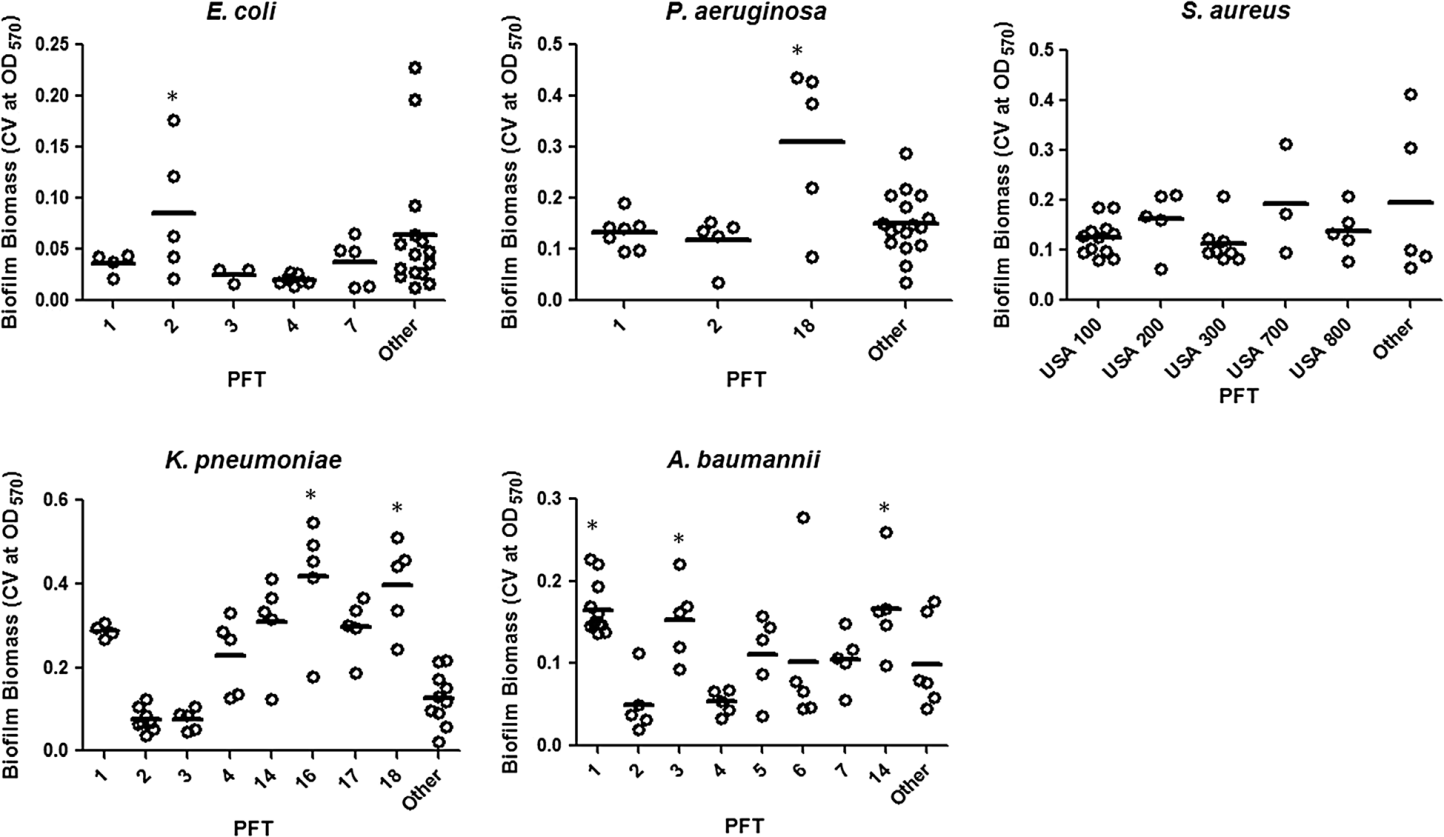
**Association between biofilm formation and pulsed-field type.** Relationship of the biofilm-forming capacity of individual strains and pulsed-field type (PFT). Data points represent the mean biofilm biomass of individual isolates tested from each of the unique PFTs, as determined by the microtiter plate assay. Line bar represents the median biofilm biomass for each PFT group. Only pulsed-field types with >3 individual strains were used for the comparison. Other denotes those groups with <3 clinical isolates. Error bars represent the standard deviation among the results for different isolates. One-Way ANOVA analysis with Holm-Sidak comparison test was used to determine statistical differences between groups. Asterisks indicate those groups that were statistically significant to the majority of PFT; *p<0.05.

Indeed, for each of the bacterial species evaluated there was a significant association between specific PFT groups and biofilm formation (Figure 3). In *E. coli* and *P. aeruginosa*, clinical isolates belonging to PFT-2 (n=5) (*p<0.001*) and PFT-18 (n=5) (*p<0.001*) groups respectively, had a significantly greater ability to form biofilms, as determined by crystal violet staining of biofilms, compared to those strains within the other PFT groups. In *S. aureus*, strains belonging to USA200 and USA 300 had a moderate, albeit insignificant, increased ability to form biofilms compared to the other PFT. In contrast, multiple PFTs in *K. pneumoniae*, including PFT-1 (n=6), 4, 14, 16, 17, and 18 (n=5), were associated with high biofilm formation of which PFT-16 (n=5) (*p*<0.01) and PFT-18 (n=5) (*p*<0.01) isolates were associated with the greatest ability to form biofilms. In contrast, those isolates belonging to PFTs 2 (n=8) and 3 (n=5) were weak biofilm forming strains. With the exception of PFTs-2 and 4 (n=5), the majority of the PFTs for *A. baumannii* were also associated with biofilm formation of which isolates from PFT-1 (n=13), 3, and 14 (n=5) (*p<0.05*) groups were associated with a greater ability to form biofilms. With the exception of a few studies evaluating the relationship of biofilm formation to *SSCmec* and *spa* typing in *S. aureus*, our study is the first to characterize and identify PFT groups associated with biofilm formation for *K. pneumoniae*, *P. aeruginosa*, and *A. baumannii*.

**Figure 4:**
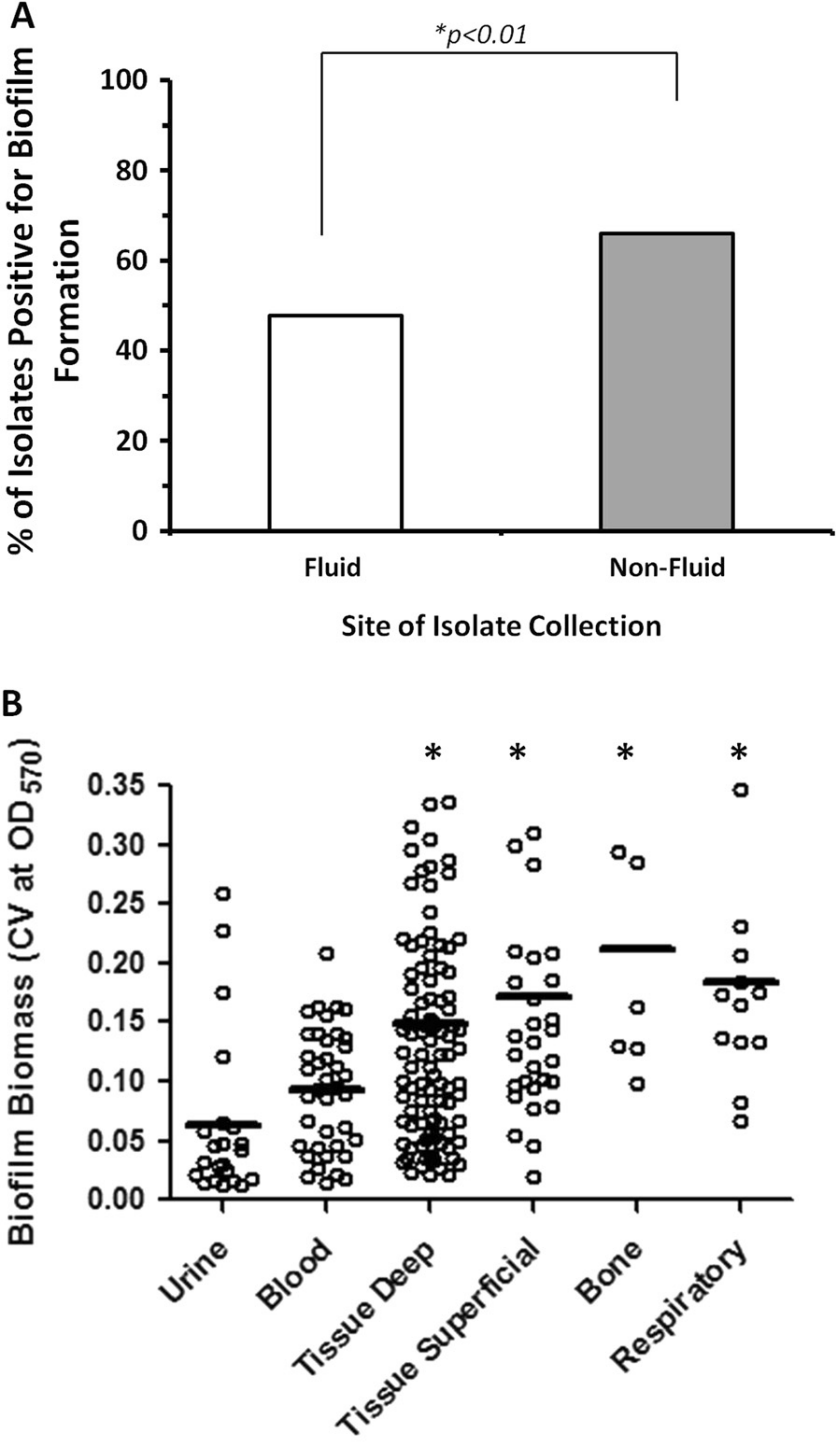
**Biofilm formation is associated with the site of isolation. A)** Distribution of biofilm-forming strains among fluid or non-fluid culture source. Bars are representative of % biofilm positive strains. Statistical analysis was performed using the *X*^2^ test. **B)** Comparison of biofilm biomass (CV_570_) from clinical isolates collected from various anatomical sites, including urine (n=27), blood (n=45), tissue deep (n=82), tissue superficial (n=31), bone (n=8) and respiratory sites (n=12), as determined by the microtiter plate assay. Data points represent the average biofilm biomass of individual isolates tested from each anatomical site and line bar represents the median biofilm biomass of the group. One-Way ANOVA analysis with Holm-Sidak comparison test was used to determine statistical differences between groups. Asterisks indicate those groups that were statistically significant; *p<0.05.

In addition to PFT, a strong correlation between biofilm formation and the site of isolate collection was observed. Clinical strains isolated from non-fluid sites including superficial/deep tissue, bone, and respiratory tract on average had a significantly higher proportion of biofilm positive strains compared to those isolates from host fluids, including blood or urine (*p=0.01*) (Figure 4A). Consistent with this, comparison of biofilm averages amongst clinical strains grouped into individual collection sites, demonstrated that isolates from superficial/deep tissue, bone, and respiratory tract (i.e. non-fluid culture sites) on average displayed enhanced biofilm formation, as determined by CV measurement, compared to those isolates from blood and urine (*p<0.01*) (Figure 4B). This may reflect adaptations favoring the survival of organisms in non-liquid physiologic environments, which may explain in part the difficulty in eradicating some infections once they become established in solid tissues. Of note, isolates from bone, including *S. aureus* and *K. pneumoniae*, had a significantly greater ability to form biofilms compared to the isolates collected from respiratory, superficial, and deep tissue groups (*p=0.04,p=0.02* and *p<0.001* respectively).

Together these findings implicate that at various anatomical sites, such as bone and soft tissue, biofilm formation may play a role in the successful colonization and/or the subsequent development of invasive disease at these particular wound sites which is partly dependent on PFT.

### Biofilm formation and multidrug-resistance

Antimicrobial resistance is an innate feature of bacterial biofilms that, in addition to the increasing rates of reported antimicrobial resistance amongst clinical strains, may further complicate patient treatment [21,38]. In comparing antimicrobial resistance to the ability of biofilm formation in the individual strains, we observed that strains capable of forming biofilms were more frequently observed to be an MDR phenotype (Table 2). In *A. baumannii*, strains capable of forming biofilms were more often observed to be resistant to aminoglycosides, carbepenems, tetracyclines, and sulfonamides compared to those strains characterized as weak biofilm producers. Similarly in *K. pneumoniae* and *S. aureus*, the ability to form biofilms was observed in strains to be resistant to cephalosporins and fluoroquinolones, respectively. Likewise, in *P. aeruginosa* strains positive for biofilm formation more commonly demonstrated a resistance phenotype to cephalosporins. Because the majority of isolates selected to evaluate biofilm formation were predominately MDR organisms, the interpretations of these results are limited. However, as previous studies have shown that biofilm formation is higher in MDR strains [20,21,39], and can promote antimicrobial resistance by selecting for highly resistant strains following treatment with sub-inhibitory antimicrobial concentrations [40,41], the ability of a strain to develop biofilms may have an important, yet not fully understood role in the development of multidrug resistance.

**Table 2:** Biofilm formation and antimicrobial resistance in clinical isolates

		***A. baumannii***		***E. coli***		***K. pneumoniae***		***P. aeruginosa***		***S. aureus***	
		**n=53**		**n=39**		**n=54**		**n=36**		**n=23**	
**% MDR**^**a**^		**86.7%; 46/53**		**79.5%; 31/39**		**100%**		**77.7%; 28/36**		**65.2%; 15/23**	
**Antimicrobial**		**%**^**b**^	**%Biofilm Production**	**%**	**% Biofilm Production**	**%**	**% Biofilm Production**	**%**	**% Biofilm Production**	**%**	**% Biofilm Production**
***Aminoglycosides***											
**Amikacin**	**R**	67	65	10	33	20	83	25	67	***ND***	***ND***
	**S**	33	47	90	15	80	50	75	81	***ND***	***ND***
**Gentamicin**	**R**	90	89	47	14	93	79	39	75	3	100
	**S**	10	67	53	19	7	71	61	59	97	90
**Tobramycin**	**R**	70	81	53	19	53	81	23	90	***ND***	***ND***
	**S**	30	33	47	14	47	64	77	78	***ND***	***ND***
***Ansamycins***											
**Rifampin**	**R**	***ND***	***ND***	***ND***	***ND***	***ND***	***ND***	***ND***	***ND***	0	92
	**S**	***ND***	***ND***	***ND***	***ND***	***ND***	***ND***	***ND***	***ND***	100	78
***Cephalosporins***											
**Cefazolin**	**R**	***ND***	***ND***	93	14	90	67	100	80	69	99
	**S**	***ND***	***ND***	7	50	10	0	0	0	31	50
**Cefepime**	**R**	***ND***	***ND***	77	9	93	82	63	79	***ND***	***ND***
	**S**	***ND***	***ND***	23	43	7	56	37	82	***ND***	***ND***
**Cefoxitin**	**R**	***ND***	***ND***	10	67	13	100	100	80	74	95
	**S**	***ND***	***ND***	90	11	87	65	0	0	26	60
**Cefotaxime**	**R**	80	63	***ND***	***ND***	93	68	***ND***	***ND***	***ND***	***ND***
	**S**	20	50	***ND***	***ND***	7	100	***ND***	***ND***	***ND***	***ND***
**Ceftazidime**	**R**	53	44	77	13	97	86	67	90	***ND***	***ND***
	**S**	47	93	23	29	3	33	33	60	***ND***	***ND***
**Ceftriaxone**	**R**	***ND***	***ND***	80	13	87	73	100	80	***ND***	***ND***
	**S**	***ND***	***ND***	20	33	13	69	0	0	***ND***	***ND***
***Carbepenems***											
**Imipenem**	**R**	67	85	0	0	0	0	75	86	***ND***	***ND***
	**S**	33	30	100	17	100	70	25	95	***ND***	***ND***
**Meropenem**	**R**	67	85	0	0	0	0	58	78	***ND***	***ND***
	**S**	33	30	100	17	100	70	42	82	***ND***	***ND***
***Flouroquinolones***											
**Ciprofloxacin**	**R**	90	85	83	12	63	79	75	80	***ND***	***ND***
	**S**	10	33	17	40	37	45	25	100	***ND***	***ND***
**Levofloxacin**	**R**	57	71	83	12	50	80	80	92	50	93
	**S**	43	92	17	40	50	60	20	50	50	80
***Penicillins***											
**Ampicillin**	**R**	100	67	97	17	100	70	100	80	100	100
	**S**	0	0	3	0	0	0	0	0	0	0
**Aztreonam**	**R**	100	67	73	9	97	90	73	73	***ND***	***ND***
	**S**	0	0	27	38	3	100	27	100	***ND***	***ND***
**Oxacillin**	**R**	***ND***	***ND***	***ND***	***ND***	***ND***	***ND***	***ND***	***ND***	83	92
	**S**	***ND***	***ND***	***ND***	***ND***	***ND***	***ND***	***ND***	***ND***	17	60
**Piperacillin**	**R**	93	71	13	0	30	78	67	75	***ND***	***ND***
	**S**	7	100	87	19	60	61	33	90	***ND***	***ND***
***Tetracyclines***											
**Tetracycline**	**R**	80	71	67	20	63	58	***ND***	***ND***	20	83
	**S**	20	50	33	10	37	91	***ND***	***ND***	80	88
***Sulfonamides***											
**Trimeth-Sulfameth**	**R**	90	78	77	17	73	86	100	80	0	0
	**S**	10	50	23	14	27	50	0	0	1000	87

^a^ A multidrug-resistant (MDR) organism was defined as any extented spectrum-lactamase (ESBL) producing bacteria, or if resistant to all tested antimicrobials on 3 or more classes of antimicrobial agent.

^b^ %; indicates the percentage of isolates resistant (R) or sensitive (S) to each antimicrobial agent. Antimicrobial suceptibilities were determined using BD Phoenix™ automated microbiology system and are reflective of clinical breakpoints.

### Biofilm formation among isolates from persistent infections

As biofilm formation has been shown to be a mechanism for evading host-defenses and resisting the effect of antimicrobials, it has been suggested that strains capable of forming biofilms may persist within the host contributing to relapsing/chronic infections [4,5,42]. Despite our understanding of biofilms, few studies have provided evidence directly implicating this phenotype in persistent infections. To examine the relationship between biofilm formation and relapsing infection, we assessed whether serial isolates from five patients with persistent infections were more likely to be positive for biofilm formation. In all five of the patients, the duration between the first and last isolate collection ranged from 0 to >100 days (Table 3). In patients 2, 3, and 5 a number of the serial isolates evaluated were collected from various sterile anatomical sites unique to site of the first isolate. With the exception of 3 of the isolates (3/34; 9%), the majority of serial isolates (31/34; 91%) from the five patients were positive for biofilm formation and was consistent between the sequential isolates even those with recovery times >100 days apart, as determined by measurement of biofilm by CV. The frequency of biofilm positive strains isolated from patients with relapsing infections is interesting and indicates a potential role for biofilm formation in these types of infection; however, future studies evaluating larger patient populations with relapsing infections and evaluating additional clinical outcomes would be necessary to fully evaluate the relationship between these two properties.

**Table 3:** Biofilm formation of serial isolates recovered from patients with clinical relapse involving clones identical to the initial isolate

**Patient**	**Bacterium**	**Isolate**	**Source**	**Days after initial isolate**	**PFT**^***a***^	**Biofilm former**^***b***^
1	*A. baumannii*	1	Deep tissue	0	1	Yes
		2	Deep tissue	3	1	Yes
		3	Bone	8	1	Yes
		4	Deep tissue	68	1	Yes
		5	Deep tissue	70	1	Yes
		6	Deep tissue	127	1	Yes
2	*P. aeruginosa*	1	Blood	0	1	Yes
		2	Blood	1	1	Yes
		3	Blood	17	1	Yes
		4	Deep tissue	21	1	Yes
		5	Pleural fluid	25	1	No
		6	Deep tissue	29	1	Yes
		7	Blood	36	1	Yes
		8	Deep tissue	42	1	Yes
		9	Respiratory tract	43	1	Yes
		10	Blood	46	1	No
		11	Respiratory tract	49	1	Yes
		12	Blood	51	1	Yes
3	*P. aeruginosa*	1	Groin swab	0	A	Yes
		2	Superficial tissue	332	B	No
		3	Superficial tissue	332	B	Yes
		4	Deep tissue	522	A	Yes
		5	Deep tissue	522	A	Yes
4	*P. aeruginosa*	1	Deep tissue	0	19	Yes
		2	Deep tissue	158	19	Yes
5	*MRSA*^*c*^	1	Deep abscess	0	USA300	Yes
		2	Deep abscess	0	USA300	Yes
		3	Superficial abscess	16	USA300	Yes
		4	Nares swab	105	USA300	Yes
		5	Nares swab	161	USA300	Yes
		6	Nares swab	202	USA300	Yes
		7	Superficial fluid	224	USA300	Yes
		8	Superficial tissue	247	USA300	Yes
		9	Deep tissue	248	USA300	Yes

^***a***^ Pulsed-field type (PFT).

^***b***^ Strains with Crystal Violet absorbance greater than or equal to that of *Staphylococcus epidermidis* ATCC 12228 (positive control) were considered to be positive for biofilm formation.

^***c***^ Methicillin-resistant *S. aureus*.

## Conclusions

We found a high prevalence of biofilm-forming phenotypes among a large number of clinical isolates representing a diversity of species, genotypes and anatomic culture sites of origin. In addition, biofilm formation was prevalent among isolates with a MDR phenotype. To our knowledge, this study is the first to demonstrate biofilm formation by serial isolates recovered from relapsing clinical infections including molecular characterizations of strain relatedness, and contributes to a limited number of studies examining biofilm formation by Gram-negative bacilli beyond the context of urinary tract infections. Our findings highlight the importance of the bacterial biofilm phenotype as a potential virulence factor which may contribute to the clinical relapse of infections.

## Competing interests

The authors declare that they have no competing interests.

## Authors’ contributions

CJS participated in the study design, experimental study and data analysis, and wrote the final draft of this manuscript. KM, MLB, and DRR performed the experimental study and acquisition of data. KM, KSA, JCW, and CKM participated in the study design, data analysis, and helped review the manuscript. All authors read and approved the final manuscript.

## Pre-publication history

The pre-publication history for this paper can be accessed here:

http://www.biomedcentral.com/1471-2334/13/47/prepub

## Supplementary Material

Additional file 1 **Literature search of previous studies evaluating biofilm formation by clinical isolates *in vitro*.** List of previous studies comparing biofilm formation by clinical isolates in various bacterial species, emphasizing the focus of study, strains and methods used to quantify biofilm formation, and conclusions drawn from studies. Click here for file
